# Prediction of Alzheimer’s Disease Based on Multi-Modal Domain Adaptation

**DOI:** 10.3390/brainsci15060618

**Published:** 2025-06-07

**Authors:** Binbin Fu, Changsong Shen, Shuzu Liao, Fangxiang Wu, Bo Liao

**Affiliations:** 1 School of Mathematics and Statistics, Hainan Normal University, Haikou 571158, China; bbf8978@163.com (B.F.); shenchangsong2019@163.com (C.S.); faw341@mail.usask.ca (F.W.); 2 Key Laboratory of Data Science and Intelligence Education, Hainan Normal University, Ministry of Education, Haikou 571158, China; 3 Zhangjiajie People’s Hospital, Zhangjiajie 427000, China; liaoshuzu123@163.com; 4 Division of Biomedical Engineering, Department of Mechanical Engineering, University of Saskatchewan, Saskatoon, SK S7N 5A9, Canada

**Keywords:** Alzheimer’s disease, domain adaptation, multi-modal data, multi-head attention mechanism

## Abstract

**Background/Objectives:** Structural magnetic resonance imaging (MRI) and 18-fluoro-deoxy-glucose positron emission tomography (PET) reveal the structural and functional information of the brain from different dimensions, demonstrating considerable clinical and practical value in the computer-aided diagnosis of Alzheimer’s disease (AD). However, the structure and semantics of different modal data are different, and the distribution between different datasets is prone to the problem of domain shift. Most of the existing methods start from the single-modal data and assume that different datasets meet the same distribution, but they fail to fully consider the complementary information between the multi-modal data and fail to effectively solve the problem of domain distribution difference. **Methods:** In this study, we propose a multi-modal deep domain adaptation (MM-DDA) model that integrates MRI and PET modal data, which aims to maximize the utilization of the complementarity of the multi-modal data and narrow the differences in domain distribution to boost the accuracy of AD classification. Specifically, MM-DDA comprises three primary modules: (1) the feature encoding module, which employs convolutional neural networks (CNNs) to capture detailed and abstract feature representations from MRI and PET images; (2) the multi-head attention feature fusion module, which is used to fuse MRI and PET features, that is, to capture rich semantic information between modes from multiple angles by dynamically adjusting weights, so as to achieve more flexible and efficient feature fusion; and (3) the domain transfer module, which reduces the distributional discrepancies between the source and target domains by employing adversarial learning training. **Results:** We selected 639 subjects from the Alzheimer’s Disease Neuroimaging Initiative (ADNI) and considered two transfer learning settings. In ADNI1→ADNI2, the accuracies of the four experimental groups, AD vs. CN, pMCI vs. sMCI, AD vs. MCI, and MCI vs. CN, reached 92.40%, 81.81%, 81.13%, and 85.45%, respectively. In ADNI2→ADNI1, the accuracies of the four experimental groups, AD vs. CN, pMCI vs. sMCI, AD vs. MCI, and MCI vs. CN, reached 94.73%, 81.48%, 85.48%, and 81.69%, respectively. **Conclusions:** MM-DDA is compared with other deep learning methods on two kinds of transfer learning, and the performance comparison results confirmed the superiority of the proposed method in AD prediction tasks.

## 1. Introduction

Alzheimer’s disease (AD) is a chronic disorder characterized by the degeneration of brain tissue [1], which will cause irreversible brain damage and seriously affect the normal operation of brain function, bringing a heavy burden to patients’ families and society. At present, the pathogenic causes of AD are complex, so there is no effective cure or drug for this disease [2]. According to the survey data of the World Health Organization (WHO), the number of dementia patients in the world has exceeded 55 million, and it is estimated that the new cases of AD are about 10 million each year [3]. Among them, mild cognitive impairment (MCI) is a key stage in the initial phase of detecting AD [4], and it is difficult to identify the characteristics of brain damage in this stage. However, some MCI can be reversed, and timely detection and intervention can delay the condition’s progression and enhance the quality of life of patients. Based on the survey, if the patients at MCI stage are not treated in time, about 80% to 90% of the patients at this stage will progress to dementia within 5 years [5]. Thus, the accurate diagnosis of early AD can identify high-risk individuals earlier, providing timely intervention and providing treatment options that may extend the life of patients.

Structural magnetic resonance imaging (MRI) can effectively capture clear structural shifts in brain atrophy, including alterations in thickness, volume, and shape [6]. 18-fluoro-deoxy-glucose positron emission computed tomography (PET) employs radioactive tracers to monitor cerebral glucose metabolism, reflect hemodynamic changes, and identify functional alterations in the brain [7]. Two types of neuroimaging, MRI and PET, are crucial for identifying AD. Due to the complicated function and structure of the brain, single-modal data cannot deliver adequate characteristic details for the diagnosis of AD. So most of the current research is based on multi-modal data experiments. However, the heterogeneity among multi-modal data shows minimal correlation, which makes it difficult to effectively use deep learning technology to integrate low-level features of different modes [8]. If only the feature fusion of multi-modal data is carried out directly, the heterogeneity between the data cannot be effectively solved, and the model will lead to poor diagnostic results for AD-related tasks.

Medical imaging data come from different devices, and there is usually inconsistency in data distribution, which is also called a domain shift issue [9]. If the domain shift issue is not solved, the performance of other domain data in the original model will be poor, which makes it difficult to apply the trained model to practical clinical practice. Traditional research directly assumes that the feature distribution among different datasets is the same. For example, Gonneaud et al. [10] sampled the neuroimaging data from multiple sites based on this assumption and directly applied the source domain-trained algorithm to the target data. However, in practical clinical applications, this assumption is not valid. Therefore, effectively solving the problem of domain migration is a key problem in the current research. As a promising approach to address domain differences [11], domain adaptive technology facilitates feature alignment by imposing constraints on data originating from disparate domains. Upon completion of training on the labeled dataset, the trained classifier can be directly deployed on the unlabeled dataset, thereby obtaining generalization performance.

### 1.1. Analysis of AD Based on Multi-Modal Data

In order to reduce the subjectivity in the diagnosis process and enhance the consistency of the results, neuroimaging technology has been progressively introduced into the research field of AD. Some research has focused on extracting features from single-modal data. Ponisio et al. [12] and Vega et al. [13] harnessed MRI and PET data to extract features for diagnosing AD and MCI, respectively. However, using the single-modal approach it is often difficult to capture the complex features of the disease comprehensively because of the limitations of the data and the complexity of the brain. Therefore, the multi-modal method has gradually become a research hotspot. For example, Yuan et al. [14] introduced a multi-modal (MRI and clinical score data) three-dimensional Inception-v4 model to diagnose AD. Yuan et al. [15] proposed that multi-modal and multi-probe PET/MR Imaging could be used for the early diagnosis of AD, and the results proved that multi-modal technology had a better effect than single mode. Liu et al. [16] proposed a multi-task and multi-modal fusion model based on hierarchical attention, and used MRI and PET data to solve the problems of multi-modal data fusion and multi-task modeling in AD diagnosis, thus boosting the classification accuracy of AD. Wang et al. [17] introduced a novel multimodality-based multiomics integration method (MOSEGCN), demonstrating that a multi-head attention mechanism can significantly enhance the classification effectiveness of AD.

These methods show good performance in dementia recognition tasks. However, the presentation of the disease and the underlying mechanisms can vary greatly from patient to patient. These methods may still not adequately address the heterogeneity of data between different modes.

### 1.2. Similarity Analysis Between Different Models

In multi-modal learning, measuring the similarity between different modes is a key step to achieving modal alignment and fusion. For example, Zhang et al. [18] proposed a multilayer graph regularization robust multimodal feature-selection method (GRMFS), which addresses the issue that the fixed affinity matrix in the traditional method only considers the similarity within the same modality while ignoring the similarity between different modalities. Lei et al. [19] developed a new framework for AD detection, which maps data from different modes into the same feature space through a feature-induced learning module to mitigate the impact of feature heterogeneity. Cheng et al. [20] used the de-correlation constraint function to reduce the feature similarity between models and enhance the ability of models to extract complementary information from different patterns. Furthermore, in the work of Liu et al. [21], a Gaussian kernel function was used to calculate the similarity between different samples. It also projects data from different views into a unified feature space through kernel canonical correlation analysis (KCCA). This approach helps to better perform representation learning and classification tasks. This method leverages the excellent mathematical properties of the Gaussian kernel function, especially in dealing with nonlinear relations, and effectively boosts the model’s performance. These studies show that dealing with the semantic differences between modes can help models better understand the intrinsic semantic association between different modal data, to effectively improve the effectiveness of multi-modal data in AD diagnosis. However, their approaches rely solely on the source data for model training while overlooking the characteristics of the target data, potentially weakening the model’s transferability.

### 1.3. Domain Adaptation Development Status

With the growing awareness of the domain shift problem in medical imaging research, domain adaptation techniques have emerged as a promising solution to address the distribution discrepancies between source and target domains. These techniques have been increasingly integrated into AD diagnosis and other medical applications.

For instance, Zhang et al. [22] proposed a passive domain adaptation framework called MPPL-SFDA, which leverages multi-center prototyping and pseudo-label strategies to achieve domain adaptation while protecting privacy. This framework significantly reduces the feature distribution differences between domains. Guan et al. [23] introduced a multi-site MRI-coordinated attention-guided deep domain adaptation structure, which is effective in automatically diagnosing neurological diseases by aligning the feature distributions across different sites. Turrisi et al. [24] demonstrated that transfer learning methods, combined with historical data and pre-trained models, can substantially mitigate domain shift issues. Dadsetan et al. [25] proposed a cross-domain self-supervised learning (CDSSL) method, which employs augmentation CDSSL and auxiliary CDSSL strategies to reduce the data variability caused by different scanners.

These studies collectively highlight the effectiveness of domain adaptation techniques in bridging the gap between source and target domains, thereby improving the generalizability and reliability of medical imaging models. Therefore, incorporating domain adaptation technology into our proposed method provides a robust foundation for addressing the domain shift problem and enhancing the performance of AD diagnosis.

### 1.4. The Present Study

**Figure 1:**
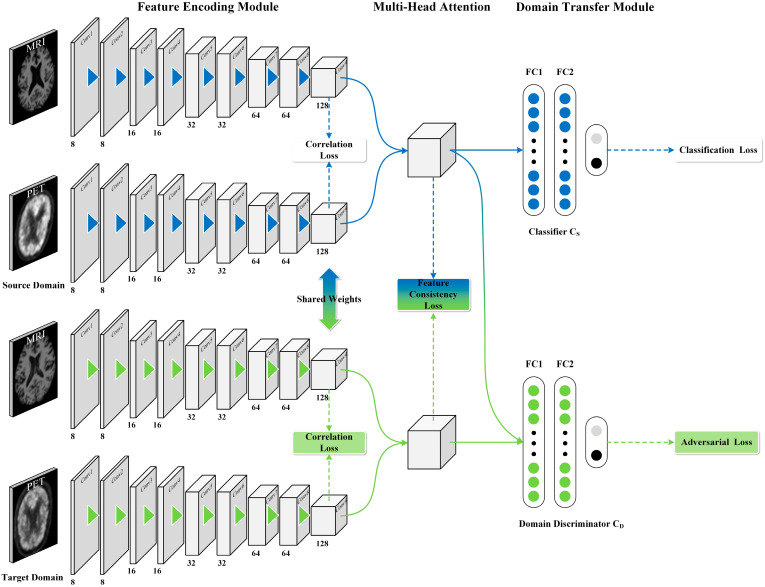
MM-DDA framework based on MRI and PET.

To address the structural and semantic differences between modes and distribution differences between two domains in AD diagnosis, we introduce a multi-modal deep domain adaptation (MM-DDA) model. As indicated in Figure 1, MM-DDA has three parts: (1) The feature extraction module, which leverages a convolutional neural network to extract feature representations from MRI and PET images in both the source and target domains. This module can efficiently address the issue of structural discrepancies between different modalities; (2) The multi-head attention fusion module fuses the extracted MRI and PET features using the multi-head attention mechanism. By doing so, complex semantic correlations between modalities can be uncovered; (3) The domain transfer module, which intends to strengthen the generalization ability of the model on the target domain by learning the shared features between the source domain and the target domain through adversarial training. In the experiment, we assessed the MM-DDA method on two independent datasets (ADNI1 and ADNI2) for multiple AD-related tasks, and the performance comparison demonstrates the superiority of this model. The contributions in this paper are as follows:

- This work presents a MM-DDA model, which fully leverages the complementary information from two modalities of data to enhance the classification accuracy. Furthermore, MM-DDA operates without requiring class labels from the target domain, thereby reducing the cost and complexity of data annotation.
- The cross-entropy loss function combined with the Gaussian kernel was employed to calculate the correlation loss between modalities, quantify and optimize the semantic similarity between different modalities, and enhance the synergy between modalities.
- This paper employs the multi-head attention mechanism to dynamically adjust the weights among different modality features and capture richer semantic information.

## 2. Materials and Methods

### 2.1. Source and Preprocessing of the Dataset

This study utilized a publicly available brain dataset, namely the Alzheimer’s Disease Neuroimaging Initiative (ADNI) [26] (https://ida.loni.usc.edu, accessed on 9 July 2024). Overall, our experiment included data from 639 ADNI1 and ADNI2 participants, and the subjects were required to have both MRI and PET images simultaneously. We downloaded the MRI and PET images of the subject within the same age group as much as possible. These 639 subjects included 279 normal cognition (CN) participants, 180 AD patients, and 180 MCI subjects. The 180 MCI subjects could be further sorted into 68 progressive MCI (pMCI) subjects and 112 stable MCI (sMCI) subjects. The category labels were obtained based on the clinical diagnoses provided by each study cohort. Based on participants’ longitudinal visits, we divided MCI subjects into sMCI (those diagnosed with MCI and not transitioned to AD within three years of follow-up) and pMCI (those diagnosed with MCI and progressing to AD within the next three years). Table 1 outlines the demographic details of the subjects in the two baseline datasets (ADNI1 and ADNI2). Age and MMSE measurements are described as mean ± standard deviation.

All T1w MRIs were preprocessed using the CAT12 (https://neuro-jena.github.io/cat/index.html#DOWNLOAD, accessed on 16 July 2024) toolkit in MATLAB R2022a, including de-noising filtering, internal resampling, bias correction and affine registration, local intensity transformation, skull stripping, spatial normalization to MNI space, and smoothing. All PET images were handled by SPM12 (https://www.fil.ion.ucl.ac.uk/spm/software/spm12/, accessed on 16 July 2024), such as head motion correction, MRI registration, min–max scaling intensity normalization, spatial normalization, and smoothing. The size of MRI and PET images after data processing was 113 × 137 × 113.

### 2.2. Problem Formulation

This experiment concentrated on the issue of label-free domain suitability for AD classification relying on MRI and PET. Let X represent the sample and Y be the corresponding category label. The source domain S obeys the distribution p, and the target domain T obeys the distribution q, but p≠q. In both S and T, each domain comprises two modalities of data. We presume that the two modal data in the source dataset conform to the distributions $p_{1}$ and $p_{2}$, respectively, and the two modal data in the target dataset conform to the distributions $q_{1}$ and $q_{2}$, respectively. Suppose that there are $N_{s}$ samples as well as the corresponding labels in the source domain, namely the $D_{S}={\left\{\left(x_{i}^{S},y_{i}^{S}\right)\right\}}_{i=1}^{N_{s}}$. while there are $N_{t}$ samples in the target domain, but no category labels, namely $D_{T}={\left\{\left(x_{j}^{T}\right)\right\}}_{j=1}^{N_{t}}$. Both domains share the same category label.

### 2.3. The Proposed Approach

#### 2.3.1. Feature Coding Module

We created a dual 3D CNN architecture to extract image features from S and T. Each domain further incorporates two sub-branches dedicated to the feature extraction from MRI and PET images. As presented in Figure 1, the feature encoding module comprises four parallel 3D CNNs. Each CNN contains nine convolutional (Conv) layers with the kernel size of 3 × 3 × 3, a stride of 1, and channel numbers of 8, 8, 16, 16, 32, 32, 64, 64, and 128. Each Conv layer is succeeded by batch normalization (BN) and leaky ReLU activation. We introduced max pooling operations with a stride of 2 in Conv2, Conv4, Conv6, and Conv8 to prevent overfitting and to enlarge the receptive field. This article does not use the padding strategy.

Gaussian nuclear energy can align the semantic features of different modes and can also maintain the similarity structure between and within modes. In addition, previous experiments have shown that the calculation of the cross-entropy loss function gradient is simple and stable, which is convenient for backpropagation and optimization [27]. Based on these motivations, we designed a cross-entropy loss function combined with Gaussian kernel to calculate the correlation loss between modes as follows:

(1) $$K\left(x_{i},x_{j}\right)=\exp\left(\frac{-{\left\|x_{i}-x_{j}\right\|}^{2}}{2\sigma^{2}}\right),$$

(2) $$L_{\mathrm{cor}}=-\sum_{i=1}^{N}\sum_{j=1}^{N}K\left(x_{i},x_{j}\right)\log\left(K\left(x_{i},x_{j}\right)+1\times 10^{-6}\right),$$

where $K\left(x_{i},x_{j}\right)$ is the value of Gaussian kernel function and $x_{i}$ and $x_{j}$ signify the feature vectors of MRI and PET images extracted after passing through nine convolutional layers. σ denotes the standard deviation of the Gaussian kernel function and is used to control the width of the Gaussian kernel function, where σ=1. $L_{\mathrm{cor}}$ is the correlation loss function. $\left(1\times 10^{-6}\right)$ is a very small constant, which prevents the input of the logarithmic function from being zero or very close to zero, thus ensuring the stability of the numerical calculation. Here, we calculated the correlation loss between MRI and PET within the S and T, namely $L_{\mathrm{cor}}^{S}$ and $L_{\mathrm{cor}}^{T}$.

#### 2.3.2. Multi-Head Attention Feature Fusion Module

The multi-head attention mechanism [28] can split the data into multiple subspaces, and each subspace independently captures the features of the data, enabling the model to understand the intrinsic information among the data from different perspectives. Based on this characteristic, the multi-head attention mechanism is selected to fuse the features in this experiment. Specifically, suppose $F_{\mathrm{MRI}},F_{\mathrm{PET}}\in {\mathbb{R}}^{B\times C\times D\times H\times W}$ are feature maps extracted from MRI and PET images, respectively, where B is the batch size, C is the number of channels, and D, H, and W are the spatial dimensions of the features. Then the MRI and PET features are stitched along the number of channels, and the stitched features are reshaped into $F_{\mathrm{reshaped}}\in {\mathbb{R}}^{B\times L\times E}$, where L=D×H×W is the sequence length and E=2C is the feature dimension. Then the reshaped features $F_{\mathrm{reshaped}}$ will be incorporated into the multi-head attention mechanism. The following describes the calculation process of $F_{\mathrm{reshaped}}$ in the multi-head attention module: (1) calculation of query, key and value:

(3) $$Q=F_{\mathrm{reshaped}}W_{Q},K=F_{\mathrm{reshaped}}W_{K},V=F_{\mathrm{reshaped}}W_{V},$$

where $W_{Q},W_{K},W_{V}$ are learnable weight matrices used to map features into the query, key, and value spaces, respectively. (2) Calculate the attention score:

(4) $$A\left(Q,K,V\right)=\mathrm{softmax}\left(\frac{QK^{\prime }}{\sqrt{d_{k}}}\right)V,$$

where $d_{k}$ is the dimension of the key vector, which is used to scale the dot product to prevent the gradient from vanishing. (3) The multi-head attention mechanism decomposes the features into multiple heads, calculates the attention of each head separately, and then concatenates the results:

(5) $$M\left(Q,K,V\right)=c\left(head_{1},head_{2},\ldots ,head_{h}\right)W_{1},$$

where $head_{i}=A\left(QW_{Q}^{i},KW_{K}^{i},VW_{V}^{i}\right)$; h is the number of heads; and $W_{1}$ is the linear transformation matrix of the output. Finally, the feature $F_{\mathrm{attn}}\in {\mathbb{R}}^{B\times L\times E}$ output by the multi-head attention module is then flattened into a one-dimensional vector $F\in {\mathbb{R}}^{d}$, where d = 256, for subsequent classification and domain adaptation tasks.

In the experiments in this paper, the number of heads for the comparisons of AD with CN, MCI with CN, AD with MCI, and pMCI with sMCI were set to 4, 4, 8, and 16, respectively. Here, we denote $F^{S}$ and $F^{T}$ as after S and T flattening, respectively. To guarantee that the target domain dataset can acquire the features from the source domain dataset as much as possible, we designed a feature consistency loss, formulated as the mean squared difference between $F^{S}$ and $F^{T}$ as follows:

(6) $$L_{\mathrm{mse}}=\frac{1}{d}{\sum_{i=1}^{d}\left(F_{i}^{S}-F_{i}^{T}\right)}^{2},$$

where d is the dimension of the flattened eigenvector; $F_{i}^{S}$ signifies the i-th element of the flattened eigenvector in S; meanwhile, $F_{i}^{T}$ indicates the i-th entry of the flattened eigenvector in T.

#### 2.3.3. Domain Transfer Module

Suppose the disparities in data features between S and T are ignored, and the data of the target domain is directly input into the trained source domain model, it may lead to a decrease in the model’s effectiveness. Therefore, we incorporated a domain adaptation module into the model. This module is made up of two components: one is a classifier, which classifies the category of the input samples; the other is a domain discriminator, which determined whether the samples come from S or T. Through co-training, the two parts can acquire a feature representation that is category discriminative and domain consistent.

The classifier $C_{S},$ is composed of three fully connected layers, having 128, 64, and 2 units. It takes the features of the source domain data after being flattened through the multi-head fusion module as input, and makes it distinguish the domain belonging of the samples as much as possible. In order to assess the discrepancy between the category label output by the classifier and the actual sample category label, we added a classification loss to the category classifier, which is defined as follows:

(7) $$L_{\mathrm{cls}}=\frac{1}{N_{S}}\sum_{i=1}^{N_{S}}L\left(C_{S}\left(x_{i}^{S}\right),y_{i}^{S}\right)=-\frac{1}{N_{S}}\sum_{k=1}^{N_{S}}{\hat{y}}_{ik}\cdot \log\left(y_{ik}\right),$$

where $N_{S}$ is the number of samples in S; $L\left(\cdot \right)$ is the cross entropy loss; $C_{S}\left(x_{i}^{S}\right)$ is the probability that the class classifier predicts the class to which the source domain sample $x_{i}^{S}$ belongs; and $y_{i}^{S}$ is the true category of the source domain sample, which is actually 1 if it belongs to the class and 0 otherwise.

The domain discriminator $C_{D},$ is composed of three fully connected layers, with the respective number of elements being 128, 64, and 2, and contains a gradient flip layer with a weight of −0.1. The flattened features of the source domain and the target domain are concatenated as the input of the discriminator. During the adversarial training process, the feature extraction module and the domain discriminator play against each other. The target domain of the feature extraction module is to generate features that can deceive the domain discriminator, so that the target domain can learn the features of the source domain as much as possible, thereby making it difficult for the domain discriminator to distinguish whether the feature comes from the source domain or the target domain. So we added a domain discrimination loss to the domain discriminator and tried to maximize it. The loss is formulated as follows:

(8) $$L_{\mathrm{dom}}=\frac{1}{N_{S+T}}\sum_{i=1}^{N_{S+T}}L\left(C_{D}\left(x_{i}\right),y_{i}^{D}\right),$$

where $N_{S+T}$ denotes the total number of samples in the training set; $L\left(\cdot \right)$ is the cross entropy loss; $y_{i}^{D}$ is the domain indicator; and $y_{i}^{D}=1$ signifies that the sample is from S while $y_{i}^{D}=0$ signifies that the sample is from T.

Our model is designed to learn features associated with disease and domain invariance in S and T. Therefore, we collectively minimized the correlation loss $L_{\mathrm{cor}}^{S}$ and $L_{\mathrm{cor}}^{T}$ for S and T in Equation (2), minimized the consistency loss $L_{\mathrm{mse}}$ in Equation (6), minimized the class classification loss $L_{\mathrm{cls}}$ in Equation (7), and optimized the domain discrimination loss $L_{\mathrm{dom}},$ in Equation (8). The total objective function of the model is constructed as follows:

(9) $$L_{\mathrm{total}}=L_{\mathrm{cor}}^{S}+L_{\mathrm{cor}}^{T}+L_{\mathrm{mse}}+L_{\mathrm{cls}}+L_{\mathrm{dom}}$$

This method can be employed to address the issue that the target domain has no label information or only a small amount of label information.

## 3. Results

### 3.1. Experimental Setup

We randomly divided the entire dataset into 70% of the training set and 30% of the test set (with a positive and negative sample ratio of approximately 0.66), and further randomly divided 30% of the data from this 70% of the training set as the validation set for parameter adjustment and model selection. We carried out the following four sets of experiments: (1) distinguishing between AD and CN, (2) distinguishing between pMCI and sMCI, (3) distinguishing between AD and MCI, and (4) distinguishing between MCI and CN. In accordance with the four groups of experiments, we consider two kinds of migration learning setups: (1) “ADNI1→ADNI2”, with ADNI1 being S and ADNI2 T; (2) “ADNI2→ADNI1”, where ADNI2 is S and ADNI1 is T.

This experiment was deployed in the Windows 10 system, with the processor being Intel(R) Xeon(R) E-2224 CPU@3.40GHz(3.41 GHz). MM-DDA is implemented with PyTorch 2.0.0. We adopted the Adam optimizer [29] to learn the network parameters, and the exponential decay rate of its first-order moment estimation and second-order moment estimation uses its default hyperparameters, namely 0.9 and 0.999. The learning rate is specified to $9\times 10^{-7}$ and the batch size is 1. In addition, to prevent overfitting, we adopted the dropout rate of 0.5.

A total of 200 epochs were trained in this paper. Firstly, 150 epochs were used to train S, that is, ① MRI and PET were input into the feature extraction module for feature extraction, and the correlation loss between MRI and PET was calculated using Equation (2). ② The extracted MRI and PET were spliced and input into the multi-head attention mechanism, and then the features output by multiple heads were flattened according to the number of channels. ③ Input the flattened features into $C_{S}$ to output the category classification results of the features, and calculate the classification loss using Equation (7). The weight parameters trained in S were shared with T. Secondly, 50 epochs were used to train both S and T simultaneously. That is, ① both S and T input MRI and PET images into the feature extraction module simultaneously to extract features, and the respective correlation losses of S and T were calculated using Equation (2). ② The extracted MRI and PET were stitched in the S and the T, respectively. Then, the stitched features of each were input into the multi-head attention mechanism. Finally, they were flattened along the number of channels, and the feature consistency loss of the flattened features in S and T is calculated by Equation (6). ③ The flattened features of the S and the T were concatenated and input into $C_{D}$ to determine the probability that the feature comes from S, and the domain discrimination loss was calculated using Equation (8). Meanwhile, the feature after flattening S was input into $C_{S}$ to determine the category of the feature, and the classification loss was calculated by using Equation (7).

Five evaluation metrics—classification accuracy (ACC), specificity (SPE) [30], sensitivity (SEN), area under the receiver operating characteristic curve (AUC), and F1 score [31]—were applied to assess the capability. TP, TN, FP, and FN were recorded as true positive, true negative, false positive, and false negative, respectively. These are specified as follows:

$$\mathrm{ACC}=\frac{\mathrm{TP}+\mathrm{TN}}{\mathrm{TP}+\mathrm{TN}+\mathrm{FP}+\mathrm{FN}},$$

$$\mathrm{SPE}=\frac{\mathrm{TN}}{\mathrm{TN}+\mathrm{FP}},$$

$$\mathrm{SEN}=\frac{\mathrm{TP}}{\mathrm{TP}+\mathrm{FN}},$$

$$F1=2\times \frac{\mathrm{Precision}\times \mathrm{Recall}}{\mathrm{Precision}+\mathrm{Recall}},$$

where $\mathrm{Precision}=\frac{\mathrm{TP}}{\mathrm{TP}+\mathrm{FP}}$, SEN is also called Recall. For each classification index, the higher their value, the better is the classification performance.

### 3.2. Comparison Methods

To gain a more comprehensive perspective, we compared the proposed MM-DDA with three deep learning-based domain adaptation methods: DAAN [32], AD^2^A [23], and PMDA [33]. Here is an introduction to these three methods.

(1) DAAN. Based on single-modal image data, DAAN achieves unsupervised domain adaptation through dynamic adversarial adaptation networks, which can obtain domain consistent features while dynamically evaluating the priority of global and local feature space layouts.
(2) AD^2^A. The AD^2^A framework combines adversarial training and attention-directed feature learning to realize automatic brain disease recognition from multi-site MRI data. The framework can automatically locate brain regions associated with disease through attention mechanisms and use adversarial learning for cross-domain knowledge transfer.
(3) PMDA. Based on the MRI data, the PMDA framework realizes the automatic assistant diagnosis of MRI data under domain bias problems by integrating multi-scale feature extraction, prototype-constrained maximum density divergence (Pro-MDD), and adversarial domain alignment.

From the AD vs. CN classification experiment in Table 2, it can be seen that on “ADNI1→ADNI2”, compared with PMDA, MM-DDA increased by 2.28%, 2.70%, 1.22%, 2.37%, and 1.30% on ACC, SEN, SPE, AUC, and F1, respectively. In “ADNI2→ADNI1”, compared with PMDA, MM-DDA increased by 3.27% in ACC, 5.01% in SEN, 3.88% in SPE, 3.67% in AUC, and 4.87% in F1. MM-DDA showed stable performance in both migration settings, with the best classification performance and less misdiagnosis and missed detection of positive and negative classes. Meanwhile, it was demonstrated that MM-DDA can effectively integrate MRI and PET features, improving the model’s ability to integrate multimodal data and thereby enhance the model’s performance.

As can be seen from the experiment of pMCI vs. sMCI in Table 3, compared with PMDA on “ADNI1→ADNI2”, MM-DDA increased by 1.05%, 6.75%, 3.54%, and 0.66% on ACC, SEN, SPE, AUC, and F1, respectively. In “ADNI2→ADNI1”, compared with PMDA, MM-DDA increased by 3.27% in ACC, 0.83% in SEN, 3.26% in AUC, and 2.10% in F1. Although, in these two migration tasks, the SPE of MM-DDA was 1.41% and 0.92% lower than that of PMDA; within an acceptable range, it can also indicate that the overall effect of MM-DDA is slightly better. In addition, as can be seen from the findings in Table 2 and Table 3, precisely identifying pMCI and sMCI is a more challenging task than recognizing AD and CN. This is because the MCI state itself is highly heterogeneous and unstable, and there are multiple potential development paths, which increases the difficulty of the task. Nevertheless, our proposed MM-DDA approach still outperforms all other methods in these more challenging tasks.

From the experiment of AD vs. MCI in Table 4, it can be seen that in “ADNI1→ADNI2”, compared with PMDA, MM-DDA increased by 4.02%, 0.98%, 3.73%, 3.66% in ACC, SEN, AUC, and F1, respectively. In “ADNI2→ADNI1”, compared with PMDA, MM-DDA increased by 4.97% in ACC, 1.95% in AUC, and 1.31% in F1. A performance index such as SEN or SPE may be lower in MM-DDA than in other methods, which indicates that other methods are better than MM-DDA in identifying negative samples. This difference may be due to the fact that MM-DDA is more focused on overall performance during the optimization process, at the expense of the ability to recognize some specific categories.

From Table 5, we can see that the five classification indexes of MM-DDA in this task classification are all optimal, which indicates that the model has significant advantages in overall classification performance, positive and negative sample recognition ability, and comprehensive balance.

## 4. Discussion

Here, we will examine the ramifications of several of the major components in the proposed MM-DDA and propose the limitations of the current work.

### 4.1. Ablation Experiment

To validate the effectiveness of each module in the developed MM-DDA model, we designed several versions of the model for ablation experiments. These versions include the following: (1) SDA, which uses only MRI data for feature extraction, domain adaptation, and classification. (2) MSDA, using MRI and PET data for feature extraction, concatenation of different modal features, and domain adaptation and classification. (3) M^3^HF, using MRI and PET data for feature extraction, multi-head fusion of different modal features, and domain adaptation and classification.

**Figure 2:**
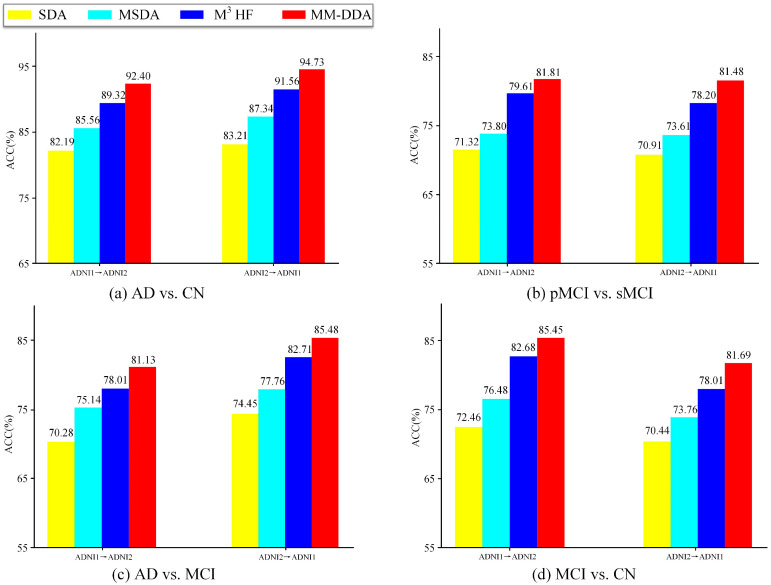
MM-DDA ablation experiment results.

Figure 2 shows the ACC values of MM-DDA with the three variants in two cross-domain tasks. First, SDA uses only MRI data for feature extraction and classification, having achieved general classification results in two cross-domain tasks. Second, when multimodal data (i.e., MSDA, using MRI and PET data) was introduced, ACC values across both domains for all tasks improved significantly, suggesting that the multimodal data itself could provide richer information. Then M^3^HF is compared with MSDA. In four groups of experiments (AD vs. CN, pMCI vs. sMCI, AD vs. MCI, MCI vs. CN), the accuracies on ADNI1→ADNI2 and ADNI2→ADNI1 are enhanced by 3.76%, 5.81%, 2.87%, 6.20%, and 4.22%, 4.59%, 4.95%, and 4.25%, respectively. This demonstrates that the multi-head attention mechanism is capable of capturing the intricate relationships between modes more effectively, thereby enhancing the model’s comprehensive understanding and processing capabilities for diverse modal data. Finally, after the introduction of inter-modal correlation loss, the efficacy of the model on all tasks is upgraded again. That is, compared to MM-DDA and M^3^HF, in the four groups of experiments, the classification tasks on ADNI1→ADNI2 and ADNI2→ADNI1 were increased by 3.08%, 2.14%, 3.12%, and 2.77%, and 3.17%, 3.28%, 2.77%, and 3.68%, respectively. This indicates that the model further learns the intrinsic correlation between MRI and PET, thereby improving the accuracy and early detection ability of AD diagnosis.

### 4.2. Determination of Head Numbers

In order to systematically evaluate the impact of multi-head model performance, this paper focuses on the first migration setting (ADNI1→ADNI2) and tests different configurations of 1, 4, 8, 16, and 32 heads on each task.

**Figure 3:**
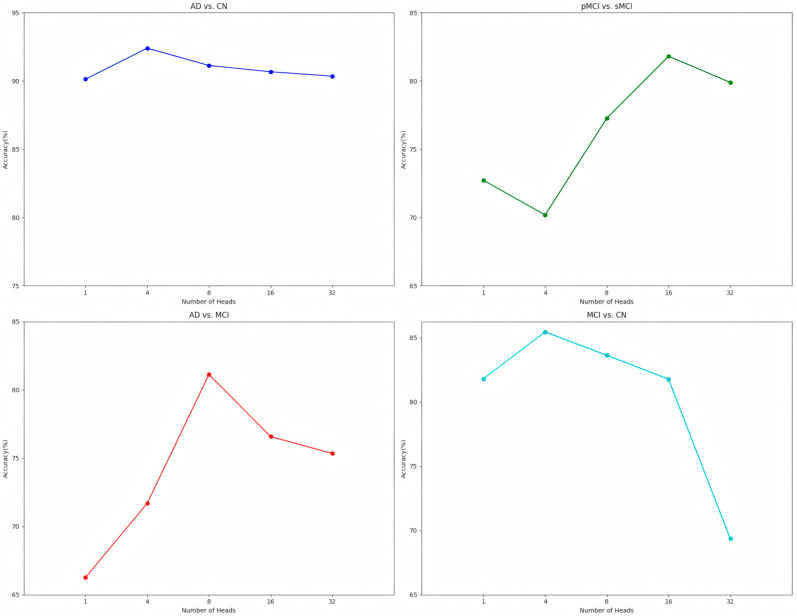
Line chart presentation of head numbers for different tasks.

Figure 3 shows the variation in accuracy rate with the number of multiple heads under different tasks. It can be seen from the figure that in AD vs. CN, the ACC value is the highest when the number of multiple heads is four. Because the complexity of this task is relatively low, the feature differences between AD and CN are obvious. A smaller number of heads can capture the key features while avoiding excessive computational complexity. In pMCI vs. sMCI, the ACC value was the highest when the number of multiple heads was 16. This task has a high complexity. The feature differences between pMCI and sMCI are subtle. More heads are needed to capture complex patterns and subtle relationships to improve the discrimination ability. Experiments verify that 16 heads achieve a better balance in performance and computing resources. In AD vs. MCI, because its task complexity is between that of pMCI vs. sMCI and AD vs. CN, the characteristic differences between AD and MCI are small and MCI is in a transitional state. The eight heads can effectively capture the complexity of this intermediate state. The experimental results show that this is the best choice. In MCI vs. CN, the complexity of this task is similar to that of AD vs. CN. Although the feature differences between MCI and CN are not as obvious as those between AD and CN, there is still a degree of discrimination. Four heads can effectively capture key features and maintain the simplicity of the model.

To sum up, based on comprehensive considerations such as the experimental results and task complexity, this paper has determined the optimal number of heads for each task. These choices can not only effectively capture the key features in the data but also avoid overfitting and unnecessary computational overhead.

### 4.3. Limitations and Prospects

The MM-DDA model focuses on binary classification tasks. Although it performs well in these specific classification tasks, there are still some limitations to be addressed. The number of samples in the dataset used by the current model is limited, the diversity of the dataset is insufficient, and the distribution of various categories is unbalanced. This may limit the generalization ability of the model.

In future work, we will collect and integrate more diverse datasets (Australian Imaging, Biomarkers and Lifestyle Studies (AIBL), Open Access Imaging Studies Series (OASIS), etc.) and incorporate some demographic information to develop models with stronger recognition and generalization capabilities. Furthermore, we will also consider extending the binary model to the multivariate model and establishing a multi-class classification model. We will also explore the interpretability of the model by introducing techniques such as SHAP or LIME, visualizing the decision-making process of the model, thereby enhancing the transparency and clinical applicability of the model.

## 5. Conclusions

This paper has developed a multi-modal deep domain adaptation (MM-DDA) model to classify the AD. Specifically, MM-DDA has three components. The first block is the feature encoding module, which serves to extract the MRI and PET features. The second block is a multi-head attention fusion module, which is implemented to combine MRI and PET features to boost the capacity of the structure for learning. The third block is the domain transfer module, which is used to transfer knowledge between domains. We have investigated the performance of MM-DDA on two benchmark datasets, ADNI1 and ADNI2. The experimental results show that compared with three methods, MM-DDA has achieved the best performance for AD classification. We carried out two migration settings on the two benchmark datasets, ADNI1 and ADNI2. In ADNI1→ADNI2, the ACC of the four groups of experiments: AD vs. CN, pMCI vs. sMCI, AD vs. MCI, and MCI vs. CN were 92.40%, 81.81%, 81.13%, and 85.45%, respectively. Similarly, in ADNI2→ADNI1, the ACC of the four groups of experiments were 94.73%, 81.48%, 85.48%, and 81.69%. Compared with the model PMDA, in ADNI1→ADNI2, the ACC of the four groups of MM-DDA experiments increased by 2.28%, 1.05%, 4.02%, and 3.89%. In ADNI2→ADNI1, the ACC of the four groups of MM-DDA experiments increased by 3.27%, 3.27%, 4.97%, and 3.12%. This indicates that the MM-DDA model in this paper has obvious advantages in dealing with multimodal data and domain adaptation tasks and this provides new tools and methods for clinical diagnosis.

## Figures and Tables

**Table 1:** Participant demographic data.

Category	Gender (F/M)	Age	MMSE
AD	70/110	75.7 ± 4.9	22.4 ± 3.2
CN	137/142	75.3 ± 5.2	29.0 ± 1.3
pMCI	31/37	76.0 ± 5.6	24.1 ± 3.9
sMCI	34/78	75.5 ± 6.8	27.6 ± 2.2

**Table 2:** AD vs. CN classification results. Bold values indicate the best performance for each evaluation metric across all methods.

S → T	Method	ACC (%)	SEN (%)	SPE (%)	AUC (%)	F1 (%)
ADNI1→ADNI2	DAAN	84.98	82.45	88.16	83.61	82.21
	AD^2^A	88.51	90.41	**90.46**	90.17	88.54
	PMDA	90.12	94.17	88.14	92.89	89.87
	**MM-DDA**	**92.40**	**96.87**	89.36	**95.26**	**91.17**
ADNI2→ADNI1	DAAN	86.23	87.51	85.46	86.55	84.71
	AD^2^A	89.16	90.66	89.14	91.61	91.44
	PMDA	91.46	87.58	92.78	91.34	89.46
	**MM-DDA**	**94.73**	**92.59**	**96.66**	**95.01**	**94.33**

**Table 3:** pMCI vs. sMCI classification results. Bold values indicate the best performance for each evaluation metric across all methods.

S → T	Method	ACC (%)	SEN (%)	SPE (%)	AUC (%)	F1 (%)
ADNI1→ADNI2	DAAN	75.43	70.17	73.14	78.51	73.55
	AD^2^A	79.24	75.36	**81.46**	86.01	**77.23**
	PMDA	80.76	78.96	81.41	85.03	74.34
	**MM-DDA**	**81.81**	**85.71**	80.00	**88.57**	75.00
ADNI2→ADNI1	DAAN	73.89	68.96	71.68	79.56	71.13
	AD^2^A	75.30	**82.17**	81.34	81.21	75.23
	PMDA	78.21	80.98	**82.17**	84.51	76.14
	**MM-DDA**	**81.48**	81.81	81.25	**87.77**	**78.24**

**Table 4:** AD vs. MCI classification results. Bold values indicate the best performance for each evaluation metric across all methods.

S → T	Method	ACC (%)	SEN (%)	SPE (%)	AUC (%)	F1 (%)
ADNI1→ADNI2	DAAN	70.56	74.38	70.01	76.18	71.21
	AD^2^A	74.77	73.87	**79.88**	80.11	74.51
	PMDA	77.11	83.86	76.26	84.27	81.18
	**MM-DDA**	**81.13**	**84.84**	75.00	**88.96**	**84.84**
ADNI2→ADNI1	DAAN	73.69	68.66	71.83	75.18	72.51
	AD^2^A	79.11	71.03	84.81	80.44	78.22
	PMDA	80.51	**74.56**	92.33	86.45	80.32
	**MM-DDA**	**85.48**	74.07	**94.28**	**88.27**	**81.63**

**Table 5:** MCI vs. CN classification results. Bold values indicate the best performance for each evaluation metric across all methods.

S → T	Method	ACC (%)	SEN (%)	SPE (%)	AUC (%)	F1 (%)
ADNI1→ADNI2	DAAN	76.31	78.21	70.45	81.07	72.78
	AD^2^A	79.21	77.48	74.44	85.11	79.01
	PMDA	81.56	84.11	82.61	86.21	81.21
	**MM-DDA**	**85.45**	**87.50**	**83.87**	**87.10**	**84.00**
ADNI2→ADNI1	DAAN	72.43	81.08	69.76	75.77	70.11
	AD^2^A	77.02	83.44	75.88	80.19	74.37
	PMDA	78.57	84.61	77.37	82.73	77.07
	**MM-DDA**	**81.69**	**86.66**	**78.05**	**85.36**	**79.93**

## Data Availability

Restrictions apply to the availability of this data. Data were obtained from the Alzheimer’s Disease Neuroimaging Initiative (ADNI) and are available at the official ADNIwebpage, https://ida.loni.usc.edu (accessed on 9 July 2024.).
